# Metformin Prescription Orders among Patients with Prediabetes in a National Network of Federally Qualified Health Centers

**DOI:** 10.1007/s11606-025-09459-w

**Published:** 2025-04-24

**Authors:** Tamkeen Khan, Lindsay Zasadzinski, Andrew Owen, Maria Vargas, Matthew O’Brien

**Affiliations:** 1 Improving Health Outcomes, American Medical Association, Chicago, IL USA; 2 Feinberg School of Medicine, Northwestern University, Chicago, IL USA; 3 Institute of Public Health and Medicine, Feinberg School of Medicine, Northwestern University, Chicago, IL USA; 4 Chicago Center for Diabetes Translation Research, Feinberg School of Medicine, Northwestern University and Pritzker School of Medicine, University of Chicago, Chicago, IL USA

**Keywords:** prediabetes, metformin, diabetes, federally qualified health centers

## Abstract

**Background:**

Clinical trials of adults with prediabetes demonstrate that metformin can prevent or delay the risk of developing type 2 diabetes by approximately 30%. The association between socioeconomic disadvantage and elevated diabetes risk underscores the importance of using metformin in this high-risk group.

**Objective:**

To examine the prevalence of metformin prescriptions among patients with prediabetes served by federally qualified health centers (FQHCs), the largest national system of primary care clinics in socioeconomically disadvantaged communities.

**Design:**

Retrospective cohort study using 2008–2019 electronic health record data from a national FQHC network.

**Participants:**

Patients with prediabetes were identified by the presence of: ≥ 1 diagnosis code; or ≥ 2 glycemic test results in the prediabetes range. We excluded patients with prior metformin prescription orders and those with prior evidence of diabetes by diagnosis code or two glycemic test results in the diabetes range.

**Main Measures:**

We examined metformin prescription orders, overall, and by patient characteristics including age and body mass index (BMI).

**Key Results:**

A total of 59,232 FQHC patients were found to have prediabetes, of whom 48.4% reported Hispanic ethnicity, 27.2% reported Black race, 22.5% had Medicaid insurance, and 33.1% were uninsured. Within one and five years of prediabetes diagnosis, metformin was prescribed for 3.0% and 6.1% of patients, respectively. In multivariate analyses, increasing BMI was the strongest predictor of metformin prescription orders. Disparities in metformin prescription rates were observed among FQHCs patients from racial minority groups relative to White patients.

**Conclusions:**

Metformin prescriptions for prediabetes are rare among FQHC patients. Prescribing rates were higher among patients with elevated BMI, and lower among patients from racial minority groups. Further research is needed to understand reasons for low metformin use in this population and promote clinical guidelines for diabetes prevention in FQHCs.

## Introduction

Prediabetes represents a high-risk state for developing type 2 diabetes (hereafter called diabetes), which is defined by elevated glycemic values that are below the diagnostic threshold for diabetes. Ninety-eight million U.S. adults (38%) have prediabetes^1^, whose annual risk of progression to diabetes has been estimated between 6 and 11%.^2,3^ Many studies have described the relationship between socioeconomic disadvantage and an elevated risk of developing diabetes.^4,5^ This association may partly explain diabetes disparities among U.S. racial and ethnic minority groups, which experience a disproportionate burden of socioeconomic disadvantage.^6^ Landmark clinical trials, including the U.S. Diabetes Prevention Program study, have demonstrated that the medication metformin lowers diabetes incidence by approximately 30% among adults with prediabetes.^7,8^ However, prior studies have found low rates of metformin prescriptions for this purpose.^9–12^ This data underscores the public health importance of metformin as an evidence-based option for diabetes prevention, especially among socially disadvantaged adults with prediabetes.

Federally qualified health centers (FQHCs) are the largest system of primary care clinics in historically disadvantaged communities, located in all 50 U.S. states and serving over 30 million patients nationwide.^13^ Over two-thirds of FQHC patients report minority race or ethnicity, 70% have household incomes below the federal poverty level, and 72% are uninsured or underinsured.^14^ The burden of diabetes is high among adult FQHC patients, with an estimated prevalence of 21% compared with 14.5% in the overall U.S. adult population.^13,15^ Further, over one-third of FQHC patients with diabetes are uncontrolled with hemoglobin A1c values greater than 9.0%.^13^ Therefore, FQHCs represent an essential setting for efforts to prevent and control diabetes among vulnerable populations, with potential to reduce observed racial and ethnic diabetes-related disparities.

The objective of this study is to examine the prevalence of metformin prescription orders for prediabetes in a large national network of adult FQHC patients. Following clinical guidelines that recommend metformin treatment especially for prediabetic adults aged < 60 years and those with class 2 or 3 obesity—groups that experienced even greater diabetes risk reductions from metformin than the overall prediabetic population—we examined prescription orders in these subgroups.^7,16^

## Methods

### Study Design and Data Source

We conducted a retrospective cohort study using electronic health record (EHR) data from a nationwide network of FQHCs located in 20 U.S. states that was available from January 1, 2008, to December 31, 2019. EHR data from primary care encounters were extracted by Alliance Chicago (Alliance), a Health Center Controlled Network that provides health information technology, clinical collaboration, and research infrastructure to its member FQHCs. For the current study, 19 participating clinic sites contributed the following EHR data from Alliance’s centralized data warehouse: sociodemographic information (sex, age, race, ethnicity, and insurance status), diagnosis codes for prediabetes and diabetes from the *International Classifications of Diseases* [Ninth revision (ICD-9) and Tenth revision (ICD-10)], glycemic laboratory test results [fasting plasma glucose (FPG), hemoglobin A1c (A1c), random blood glucose (RBG)], physical measurements (height in meters and weight in kilograms used to calculate BMI), pregnancy status, metformin prescription orders, and dates of service.

### Participants

The study included adult FQHC patients aged 18 years or older with prediabetes, defined by: 1) the presence of a diagnosis code (i.e., ICD-9 codes 790.x or ICD-10 codes R73.x); or 2) two glycemic test results in the prediabetes range (i.e., FPG: 100–125 mg/dL or A1c: 5.7–6.4%) within a two-year period.^17^ We excluded patients with prior evidence of diabetes, defined by 1) the presence of a diagnosis code (i.e. ICD-9 codes 250.x or ICD-10 codes E11.x); or 2) two glycemic test results in the diabetes range (i.e., FPG: ≥ 126 mg/dL, A1c ≥ 6.5%) within a two-year period. These laboratory criteria are derived from a validated EHR phenotype for diabetes.^18^ The same criteria were used to ascertain the development of diabetes among study cohort during the follow-up period. We also excluded all patients with any prior metformin prescription orders before clinical and/or lab diagnosis, as well as those with evidence of pregnancy during the study period. Patients with at least one follow-up visit documented within five years were analyzed for receipt of metformin prescription.

### Key Variables

The primary outcome, metformin prescription orders, were ascertained using EHR data that included the medication name and prescription order date.

To examine prediabetic subpopulations that should be prioritized for metformin treatment according to clinical practice guidelines that have been in place since the beginning of the study period, we examined age (dichotomized by age < 60 years vs. ≥ 60 years) and BMI.^16^ BMI values were computed using clinic-measured weight in kilograms and height in meters (kg/m^2^), which were ascertained on the office visit closest to prediabetes diagnosis (within 1 year). Weight status was analyzed according to the following standardized BMI cutoffs: underweight (BMI < 18.5 kg/m^2^), normal weight (BMI 18.5–24.9 kg/m^2^), overweight (BMI 25–29.9 kg/m^2^), class 1 obesity (BMI 30–34.9 kg/m^2^), and class 2 or 3 obesity (BMI ≥ 35 kg/m^2^). For Asian patients, weight status was classified based on Asian-specific BMI cutoffs recommended by experts, namely: underweight (BMI < 18.5 kg/m^2^), normal weight (BMI 18.5–22.9 kg/m^2^), overweight (BMI 23.0–24.9 kg/m^2^), class 1 obesity (BMI 25–29.9 kg/m^2^), and class 2 or 3 obesity (BMI ≥ 30 kg/m^2^).^19^ Patients without any information on race or ethnicity were considered as “unknown”. Those who were prescribed metformin and had at one least follow-up visit within the five years of prediabetes diagnosis were included in analysis.

### Statistical Analysis

Descriptive statistics were used to characterize the study population with respect to sociodemographic and clinical factors. We used Chi-square tests to examine the difference in patient characteristics between those who received a metformin prescription order compared with those who did not. We also examined receipt of metformin prescription orders within one and five years among all patients in this prediabetic cohort, and separately within subgroups defined by age (< 60 years) and BMI (class 2 or 3 obesity). Lastly, we utilized logistic regression to model metformin prescriptions among patients who had not developed prediabetes within the five years of diagnosis. The model was adjusted for race, ethnicity, sex at birth, age, BMI status, prediabetes identification criteria, and insurance type within the five-year period after diagnosis. All analyses were conducted using SAS 9.4 (SAS Institute Inc. Cary, NC) and STATA 17 (StataCorp LP, College Station, TX). For all statistical testing, significance was defined by a p-value < 0.05. The study protocol was approved by Northwestern University’s Institutional Review Board (IRB).

## Results

**Figure 1:**
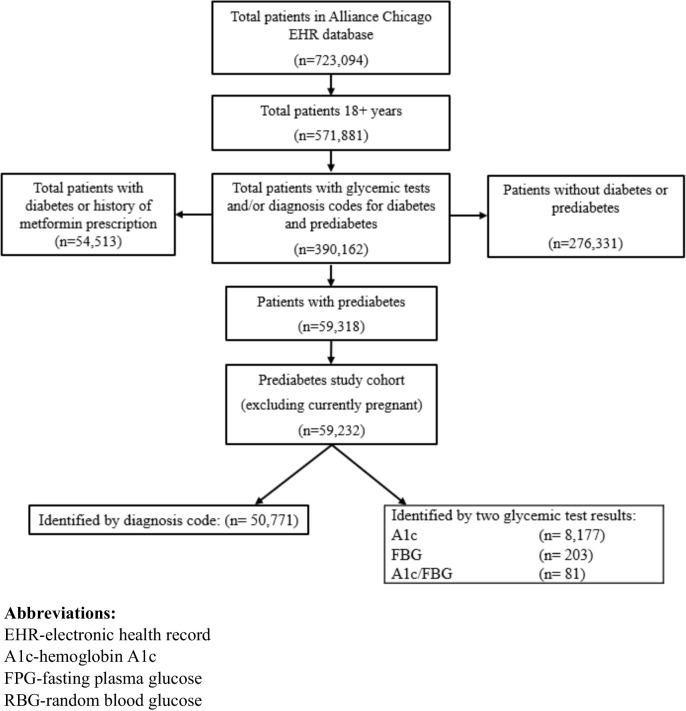
Study flow diagram

Figure 1 shows a total of 59,232 patients with prediabetes were included in the study cohort. Table 1 presents the sociodemographic and clinical characteristics of the study population. The mean age of the cohort was 48 years (± 13.5) years and approximately two-thirds of patients were women. Over one-quarter of patients reported Black race and almost half reported Hispanic or Latino ethnicity. Approximately half of the patients were either uninsured or had Medicaid insurance. Only 13.7% had private health insurance and 6.8% had Medicare coverage. Insurance status was not known for approximately one-quarter of patients. Most members of the study cohort were overweight or obese (88.5%), with the following distribution of weight categories: overweight (27.8%), class 1 obesity (29.3%), and class 2 or 3 obesity (31.4%). A total of 5,314 (9.0%) of patients developed diabetes (identified by diagnosis code or two glycemic tests) during the 5-year study period.

**Table 1:** Characteristics of FQHC Patients with Prediabetes

Total Sample	N (%)
Identification criteria	59,232
ICD-code	50,771 (85.7)
2 confirmatory labs	8,461 (14.3)
Sex^a^	
Male	21,290 (35.9)
Female	37,935 (64.0)
*Mean age, years (SD)*	*48.0 (13.5)*
Age	
18–29 Years	6,067 (10.2)
30–39 Years	11,272 (19.0)
40–49 Years	14,591 (24.6)
50–59 Years	15,009 (25.3)
60 + Years	12,293 (20.8)
Race	
White	29,359 (49.6)
Black	16,119 (27.2)
Asian	2,536 (4.3)
Other	1,164 (2.0)
Unknown	10,054 (17.0)
Ethnicity	
Hispanic	28,590 (48.4)
Non-Hispanic	28,729 (48.6)
Unknown	1,797 (3.0)
*Mean BMI, kg/m*^*2*^* (SD)*^*a*^	*32.6 (7.7)*
Weight Status	
Underweight	282 (0.5)
Normal	6,427 (11.0)
Overweight	16,172 (27.8)
Class 1 obese	17,077 (29.3)
Class 2 or 3 obese	18,311 (31.4)
Insurance	
Uninsured	19,588 (33.1)
Unknown	14,183 (24.0)
Medicaid	13,296 (22.5)
Private	8,107 (13.7)
Medicare	4,051 (6.8)
Progression to Diabetes (n = 5,997)	
< 1 year	1,955 (32.6)
1–3 years	2,420 (40.4)
3–5 years	939 (15.7)
5 + years	683 (11.4)

^a^Missing data: *n* = 7 unknown sex; *n* = 963 weight status missing

*Abbreviations*: BMI, body mass index; FQHC, federally qualified health center; ICD, International Classification of Diseases, 9th or 10th Revision

We ascertained metformin prescriptions among patients who had at least one follow-up visit within five years and did not develop diabetes during this time (Table 2). Among the 53,666 patients meeting these criteria, only 3,259 (6.1%) received a metformin prescription. Approximately two-thirds of those prescribed metformin were female (67.7%) or between the ages of 30–59 years (68.3%). Among those who received a metformin prescription, 32.7% were aged 18–39 years compared with 30.1% of those who did not receive a metformin prescription.

**Table 2:** Characteristics of Eligible FQHC Patients with Prediabetes by Metformin Prescription Receipt Within 5 Years of Prediabetes Diagnosis

	Overall N (%)	Received Metformin Prescription N (%)	Did Not Receive Metformin Prescription N (%)	Chi-Square *p*-value
	53,666	3,259 (6.1)	50,407 (93.9)	
Identification Criteria				
ICD code	48,785 (90.9)	2,916 (89.5)	45,869 (91.0)	.003
2 confirmatory labs	4,881 (9.1)	343 (10.5)	4,538 (9.0)	
Sex^a,b^				
Male	19,185 (35.7)	1,051 (32.3)	18,134 (36.0)	< .001
Female	34,474 (64.2)	2,207 (67.7)	32,267 (64.0)	
*Mean age in years (SD)*	*47.2(13.4)*	*46.5 (13.6)*	*47.2 (13.4)*	
Age				
18–29 Years	5,774 (10.8)	418 (12.8)	5,356 (10.6)	< .001
30–39 Years	10,489 (19.5)	647 (19.9)	9,842 (19.5)	
40–49 Years	13,363 (24.9)	750 (23.0)	12,613 (25.0)	
50–59 Years	13,422 (25.0)	828 (25.4)	12,594 (25.0)	
60 + Years	10,618 (19.8)	616 (18.9)	10,002 (19.8)	
Race				
White	26,745 (49.8)	1,869 (57.3)	24,876 (49.4)	< .001
Black	14,500 (27.0)	870 (26.7)	13,630 (27.0)	
Asian	2,260 (4.2)	109 (3.3)	2,151 (4.3)	
Other	1,018 (1.9)	40 (1.2)	978 (1.9)	
Unknown	9,143 (17.0)	371 (11.4)	8,772 (17.4)	
Ethnicity				
Hispanic	26,220 (48.9)	1,563(48.0)	24,657 (48.9)	0.277
Non-Hispanic	25,688 (47.9)	1,599 (49.1)	24,089 (47.8)	
Unknown	1,648 (3.1)	90 (2.8)	1,661 (3.3)	
*Mean BMI, kg/m*^*2*^* (SD)*^*b*^	*32.5 (7.6)*	*35.6 (8.5)*	*32.3 (7.5)*	
Weight status				
Underweight	272 (.5)	5 (0.2)	267 (0.5)	< .001
Normal	6,022 (11.4)	161 (5.0)	5,861 (11.8)	
Overweight	14,972 (28.3)	647 (20.1)	14,325 (28.8)	
Class 1 obese	15,506 (29.3)	906 (28.2)	14,600 (29.3)	
Class 2 or 3 obese	16,210 (30.6)	1,495 (46.5)	14,715 (29.6)	
Insurance type				
Uninsured	18,078 (33.7)	1,131 (34.7)	16,947 (33.6)	< .001
Unknown	12,559 (23.4)	609 (18.7)	11,950 (23.7)	
Medicaid	12,292 (22.9)	781 (24.0)	11,511 (22.8)	
Medicare	3,326 (6.2)	223 (6.8)	3,103 (6.2)	
Private	7,411 (13.8)	515 (15.8)	6,896 (13.7)	

^a^Missing data: *n* = 7 unknown sex; *n* = 684 weight status missing

^**b**^Chi-square analysis does not include “unknown” category for sex and weight status

*Abbreviations*: BMI, body mass index

Almost all patients prescribed metformin were overweight or obese (94.8%), and prescription rates were highest in those with class 3 obesity (46.5%). Among patients who did not receive a metformin prescription order, the overall proportion with overweight/obesity and those with class 3 obesity was comparatively smaller (87.7% and 29.6%, respectively). White patients constituted 49.8% of the sample and accounted for 57.3% of patients who received a metformin prescription. The bivariate association of patient characteristics with metformin prescription status was significant for every variable except ethnicity (*p* = 0.28).

Overall, 3.0% and 6.1% of FQHC patients with prediabetes received a metformin prescription within one and five years of their prediabetes identification, respectively (Table 3). At each of these same time intervals, the rates of metformin prescribing were nearly identical among patients younger than age 60 years, who represent a priority subgroup for metformin treatment according to clinical guidelines. Among those with class 2 and 3 obesity, another priority population for metformin treatment, prescription rates were higher (i.e. 4.4% within one year and 9.2% within five years).

**Table 3:** Receipt of Metformin Prescriptions among Patients with No Diabetes Diagnosis within 1 and 5 Years, According to Age and Class 2 or 3 Obesity

	No Diabetes Diagnosis < 1 Year N	Metformin Prescription < 1 Year N (%)	No Diabetes Diagnosis < 5 Years N	Metformin Prescription < 5 Years N (%)
Total	57,014	1,699 (3.0)	53,666	3,259 (6.1)
Age < 60 years	47,797	1,368 (2.9)	43,048	2,643 (6.1)
Class 2 or 3 obese	17,484	770 (4.4)	16,210	1,495 (9.2)
Age < 60 years & Class 2 or 3 obese	14,980	682 (4.5)	13,974	1,320 (9.4)

All patients with prediabetes had at least one follow up visit within 5 years of diagnosis

Multivariable analyses confirmed the association between patients’ weight and metformin prescriptions. Relative to patients with prediabetes and normal weight, greater multivariable odds of metformin prescription were observed among those with overweight (OR:1.66 [CI: 1.39–1.98]), class 1 obesity (OR: 2.29 [CI:1.93–2.72]), and class 2 or 3 obesity (OR:3.75 [CI: 3.18–4.44]). Patients who reported Black (OR: 0.76 [CI: 0.69–0.85]), Asian (OR: 0.65 [CI: 0.52–0.80]), Other (OR: 0.49 [CI: 0.35–0.68]) or Unknown (OR: 0.54 [CI: 0.48–0.61]) race exhibited lower multivariable odds of receiving metformin prescription orders compared with White patients. Females were more likely to be prescribed metformin than male patients (OR: 1.09 (CI:1.00–1.18) (Table 4).

**Table 4:** Logistic Regression of Metformin Prescription among Patients with No Diabetes Diagnosis within 5 years

	OR (95%CI)
Identification Criteria	
ICD code	0.84 (0.74–0.95)**
2 confirmatory labs	Ref
Sex	
Female	1.09 (1.00–1.18)*
Male	Ref
Age	
18–29 Years	1.08 (0.94–1.24)
30–39 Years	0.94 (0.83–1.06)
40–49 Years	0.88 (0.79–0.99)
50–59 Years	1.04 (0.92–1.16)
60 + Years	Ref
Race	
White	Ref
Black	0.76 (0.69–0.85)**
Asian	0.65 (0.52–0.80)**
Another	0.49 (0.35–0.68)**
Unknown	0.54 (0.48–0.61)**
Ethnicity	
Hispanic	1.00 (0.90–1.11)
Non-Hispanic	Ref
Unknown	0.95 (0.75–1.19)
Weight status	
Underweight	0.65 (0.26–1.59)
Normal	Ref
Overweight	1.66 (1.39–1.98)**
Class 1 obese	2.29 (1.93–2.72)**
Class 2 or 3 obese	3.75 (3.18–4.44)**
Insurance type	
Uninsured	1.05 (0.95–1.17)
Unknown	0.73 (0.65–0.82)**
Medicaid	Ref
Medicare	1.12 (0.95–1.32)
Private	1.11 (0.98–1.25)

^*^ *p* < 0.05

^**^p < .001

## Discussion

To our knowledge, this is the first study examining metformin prescriptions for diabetes prevention in FQHCs. Our study found that only 6.1% of FQHC patients with prediabetes received a metformin prescription within five years of prediabetes identification. Although this rate is similar to population-based estimates^9–12^, FQHCs manage a disproportionate burden of diabetes risk among its vulnerable patient population. Metformin prescribing was significantly higher among patients with overweight or obesity, relative to patients with normal weight. We did not find higher rates of metformin prescription orders among patients younger than age 60, another group for whom clinical guidelines recommend metformin for diabetes prevention. This study documents unfortunate racial disparities in metformin prescribing among patients from racial minority groups. Our findings document infrequent metformin use in a particularly vulnerable FQHC patient population, where an effective, safe, and inexpensive medication could represent an important strategy for diabetes prevention on a national scale.

A large body of public health research has documented an association between socioeconomic disadvantage and increased diabetes risk. This observation is especially relevant to prediabetes management in FQHCs, where 70% of patients receiving ambulatory care have household incomes below the federal poverty level.^13^ Many other social determinants of diabetes risk also disproportionately impact adults with low household incomes^20^, including limited educational attainment^21^, food insecurity^22^, housing instability^23^, and shift work with long working hours.^23^ As the primary system of ambulatory care for patients experiencing these exposures across the U.S., FQHCs offer diverse services to address social needs.^24^ However, the impact of these wrap-around social services on diabetes prevention is not known.

Despite promising findings about the effectiveness of metformin for preventing or delaying diabetes, estimates of its use among adults with prediabetes remain very low. Studies before 2012 indicated that metformin was taken by less than 1% of adults with prediabetes, as determined by analyses of nationally representative survey data and EHR data from a large health system.^25,26^ Recent studies of national survey data and insurance claims report more frequent use of metformin in this population, ranging from 2.0% to 8.3% of adults with prediabetes.^27,28^ Analyses of survey data generally found higher rates of metformin use than studies using EHR data or pharmacy claims, suggesting a potential recall bias with self-reported data. None of these prior studies have focused on metformin use among socioeconomically disadvantaged adults with prediabetes, who have an especially high risk of developing diabetes.

The landmark Diabetes Prevention Program (DPP) clinical trial identified prediabetic subgroups who may derive the greatest benefit from metformin treatment, including adults with class 2 or 3 obesity and adults younger than 60 years old. We found that metformin prescriptions were more common among FQHC patients with class 2 or 3 obesity, which is also supported by clinical guidelines from the American Diabetes Association (ADA) to prioritize metformin treatment in this group.^16^ Recent studies in other settings have also reported that patients with prediabetes and BMI ≥ 35 kg/m^2^ were more likely to receive metformin prescriptions than those in lower BMI categories.^27,28^ Another prediabetic subpopulation for whom ADA recommends metformin treatment is adults younger than 60 years old. We did not find a higher rate of metformin prescribing among this subpopulation, relative to older adults. Other recent studies have not reported significantly increased metformin use among younger adults with prediabetes.

The landmark DPP trial also found that Black adults experienced a greater reduction in diabetes incidence with metformin (i.e., 44%) than those observed in other racial or ethnic groups: i.e., White (24%); Hispanic (31%); American Indian (25%); and Asian (38%).^7^ Health equity experts advocate against making clinical guidelines based on race^29^; and accordingly, ADA does not recommend prioritizing metformin treatment for Black patients with prediabetes.^16^ However, our analysis documents unfortunate racial disparities in metformin prescription orders, with the highest rates observed among White FQHC patients. Future research is needed to understand the underlying causes of these racial disparities and to develop interventions to increase metformin prescribing in this setting. One prior study examined the effectiveness of an EHR-based clinical decision support tool promoting metformin prescriptions for prediabetes in a Midwestern FQHC. Use of this decision support tool doubled the rate of metformin prescribing; however, this increase did not achieve statistical significance (*p* = 0.06).^30^

There are many barriers to prescribing metformin for diabetes prevention. First, the Federal Drug Administration (FDA) has not approved this indication for metformin treatment, requiring providers to prescribe metformin as an “off label” treatment for prediabetes.^31^ Further, prior research has demonstrated that clinicians have limited awareness about recommendations to use metformin for prediabetes management. A recent national survey of primary care providers found that only 17.8% of primary care providers knew that this was a recommended initial treatment for prediabetes.^32^ In the same survey, many more clinicians expressed concern about potential side effects of metformin (74.0%) and the potential concern for renal complications in patients with chronic kidney disease (54.4%).^32^ Clinicians have also expressed skepticism about metformin’s benefits among patients with prediabetes.^31^ Prior research has also identified patient-level barriers to taking metformin for diabetes prevention, most commonly concern about potential side effects and the desire to attempt healthy lifestyle changes before medication therapy.^33^

Findings from the current analysis and studies from other clinical settings suggest that 20-year-old evidence on the effectiveness of metformin for preventing diabetes has not been translated successfully into real-world clinical practice. Low uptake of metformin for this purpose is especially concerning along with low adoption of intensive lifestyle interventions for diabetes prevention found in other studies^30^, suggesting a significant missed opportunity to use pharmacological interventions to prevent diabetes among those at highest risk. Future studies should examine use of semaglutide and tirzepatide among FQHC patients with prediabetes, as these medications recently demonstrated much greater efficacy for diabetes prevention than metformin.^34,35^ Given their high cost, it is likely that uptake of these newer medications will be slow among FQHC patients.

### Strengths and Limitations

This study is the first to examine metformin prescriptions for prediabetes in FQHCs, the largest network of primary care clinics serving socioeconomically disadvantaged patients. Our unique study population represents a significant strength, as well an opportunity to address diabetes health equity among the vulnerable populations served by FQHCs. Having five years of follow-up data enabled a longitudinal examination of metformin prescribing in this setting; whereas, prior studies were either cross-sectional or included a shorter follow-up time.^9,10,25–28^ This is particularly important because many patients with prediabetes do not have frequent primary care visits, and competing clinical demands often hinder the ability to address prediabetes during brief office visits.^31,32^Another strength is defining prediabetes by diagnosis codes or glycemic tests results. Prior research documents that prediabetes diagnosis codes are not uniformly used in primary care practice.^36,37^

Our study also has notable limitations. We used data from 2008–2019 and a 5-year follow-up period to maximize ascertainment of metformin prescriptions, given many short-term challenges that can impact prescribing patterns, including infrequent office visits. In some health systems, patients may be classified as a new patient after three years without a follow-up visit. However, this did not impact our findings because we had unique patient identifiers that maintained patient follow-up throughout the entire study period. Our approach did not explore changes in practice patterns or other factors that can influence metformin prescribing during this long time-period. We could not measure treatment adherence using our EHR data source. We were also unable to examine patients’ refusal of metformin after their providers’ recommendation for this treatment. Qualitative research is needed to explore discordance between providers’ recommendations and patients’ acceptance of metformin prescriptions. Additionally, we did not have household income data to measure socioeconomic status. FQHCs represent the largest system of care for socioeconomically disadvantaged patients nationwide, which by nature constrains variability in socioeconomic status across our study cohort. Further, high rates of uninsured patients (i.e., 32.6%) and those with Medicaid coverage (i.e., 22.0%) provide indirect evidence of their limited socioeconomic status. Infrequent use of diagnostic codes for gestational diabetes hindered analysis of women with this condition, Finally, participation in lifestyle change programs was not available in our EHR data source, precluding analysis of the other evidence-based treatment for prediabetes.

## Conclusions

While metformin is a safe, effective, and inexpensive medication for preventing diabetes, our study suggests that it is infrequently used for this purpose in FQHCs, and less so among patients from racial minority groups. We found that metformin was more commonly used for diabetes prevention among women patients with class 2 or 3 obesity. Future research should explore strategies to increase metformin prescribing rates among FQHC patients with prediabetes. This will likely require addressing barriers among providers and patients, as well as potential system-level challenges in FQHCs and regulatory policies governing metformin’s use for diabetes prevention.

## Data Availability

Those interested in accessing the study data should contact the corresponding author.

## Declarations:

### Permission to Adapt or Reuse any Copyrighted Material:

No copyrighted material, surveys, instruments, or tools were used in the research described in this article.

## References

[1] Centers for Disease Control and Prevention. National Diabetes Statistics Report website. Published 2023. Accessed 25 Nov 2023.

[2] Diabetes Prevention Program Research Group. 10-year follow-up of diabetes incidence and weight loss in the Diabetes Prevention Program Outcomes Study. Lancet. 2009;374(9702):1677-1686. 19878986 10.1016/S0140-6736(09)61457-4 PMC3135022

[3] **Yeboah J, Bertoni AG, Herrington DM, Post WS, Burke GL.** Impaired fasting glucose and the risk of incident diabetes mellitus and cardiovascular events in an adult population: MESA (Multi-Ethnic Study of Atherosclerosis). J Am Coll Cardiol. 2011;58(2):140-146. 21718910 10.1016/j.jacc.2011.03.025 PMC3146297

[4] **Beckles GL.** Disparities in the prevalence of diagnosed diabetes—United States, 1999–2002 and 2011–2014. MMWR Morb Mortal Wkly Rep*.* 2016;65. 10.15585/mmwr.mm6545a4 27855140

[5] **Bonilla GS, Rodriguez-Gutierrez R, Montori VM.** What we don’t talk about when we talk about preventing type 2 diabetes—addressing socioeconomic disadvantage. JAMA Intern Med*.* 2016;176(8):1053-1054. 27367180 10.1001/jamainternmed.2016.2952

[6] KFF. Poverty Rates by Race/Ethnicity. https://www.kff.org/other/state-indicator/poverty-rate-by-raceethnicity/. Published 2024. Accessed 6–11–2024 2024.

[7] **Knowler WC, Barrett-Connor E, Fowler SE, et al.** Reduction in the incidence of type 2 diabetes with lifestyle intervention or metformin. 2002. 10.1056/NEJMoa012512 PMC1370926 11832527

[8] **Moin T, Schmittdiel JA, Flory JH, et al.** Review of metformin use for type 2 diabetes prevention. Am J Prev Med*.* 2018;55(4):565-574. 30126667 10.1016/j.amepre.2018.04.038 PMC6613947

[9] **Moin T, Li J, Duru OK, et al.** Metformin prescription for insured adults with prediabetes from 2010 to 2012: a retrospective cohort study. Ann Intern Med*.* 2015;162(8):542-548. 25894024 10.7326/M14-1773 PMC4682357

[10] **Hughes A, Khan T, Kirley K, et al.** Metformin prescription rates for patients with prediabetes. J Am Board Fam Med*.* 2022;35(4):821-826. 35896449 10.3122/jabfm.2022.04.210485

[11] **Speaker SL, Rastogi R, Sussman TA, Hu B, Misra-Hebert AD, Rothberg MB.** Treatment of patients with prediabetes in a primary care setting 2011–2018: an observational study. J Gen Intern Med*.* 2021;36:923-929. 33449282 10.1007/s11606-020-06354-4 PMC8041989

[12] **Wu J, Ward E, Threatt T, Lu ZK.** Metformin prescribing in low-income and insured patients with prediabetes. J Am Pharm Assoc*.* 2017;57(4):483-487. 10.1016/j.japh.2017.04.008 28551306

[13] Health Resources and Services Administration. HRSA Health Center Program. https://bphc.hrsa.gov/about-health-center-program. Accessed 6–11–2024 2024.

[14] National Association of Community Health Centers. Community Health Centers: Providers, Partners and Employers of Choice. 2024.

[15] **Cheng YJ, Kanaya AM, Araneta MRG, et al.** Prevalence of diabetes by race and ethnicity in the United States, 2011-2016. Jama*.* 2019;322(24):2389-2398. 31860047 10.1001/jama.2019.19365 PMC6990660

[16] American Diabetes Association. 3. Prevention or delay of diabetes and associated comorbidities: standards of care in diabetes—2024. Diabetes Care*.* 2024;47(Supplement_1):S43-S51. 10.2337/dc24-S003 PMC10725807 38078581

[17] **O’Brien MJ, Bailey SC, Gregory DL, et al.** Screening for Prediabetes and Diabetes in a National Network of Federally Qualified Health Centers: An Observational Study. J Gen Intern Med*.* 2023;38(16):3541-3548. 37731136 10.1007/s11606-023-08402-1 PMC10713898

[18] **Nichols GA, Desai J, Lafata JE, et al.** Construction of a multisite DataLink using electronic health records for the identification, surveillance, prevention, and management of diabetes mellitus: the SUPREME-DM project. Prev Chronic Dis*.* 2012;9. 10.5888/pcd9.110311 PMC3457753 22677160

[19] World Health Organization. The Asia-Pacific Perspective: Redefining Obesity and its Treatment. 2000.

[20] **Hill-Briggs F, Adler NE, Berkowitz SA, et al.** Social determinants of health and diabetes: a scientific review. Diabetes Care*.* 2021;44(1):258. 10.2337/dci20-0053 PMC7783927 33139407

[21] **Fleischer NL, Henderson AK, Wu Y-H, Liese AD, McLain AC.** Disparities in diabetes by education and race/ethnicity in the US, 1973–2012. Am J Prev Med*.* 2016;51(6):947-957. 27554365 10.1016/j.amepre.2016.06.019

[22] **Walker RJ, Williams JS, Egede LE.** Pathways between food insecurity and glycaemic control in individuals with type 2 diabetes. Public Health Nutr*.* 2018;21(17):3237-3244. 30088467 10.1017/S1368980018001908 PMC6258341

[23] **Shan Z, Li Y, Zong G, et al.** Rotating night shift work and adherence to unhealthy lifestyle in predicting risk of type 2 diabetes: results from two large US cohorts of female nurses. BMJ*.* 2018;363. 10.1136/bmj.k4641 PMC6247172 30464025

[24] **Lewis JH, Whelihan K, Navarro I, Boyle KR, Team SCSI.** Community health center provider ability to identify, treat and account for the social determinants of health: a card study. BMC Fam Pract*.* 2016;17:1-12. 27567892 10.1186/s12875-016-0526-8 PMC5002327

[25] **Schmittdiel JA, Adams SR, Segal J, et al.** Novel use and utility of integrated electronic health records to assess rates of prediabetes recognition and treatment: brief report from an integrated electronic health records pilot study. Diabetes Care*.* 2014;37(2):565-568. 24271190 10.2337/dc13-1223 PMC3898765

[26] **Tseng E, Yeh H-C, Maruthur NM.** Metformin use in prediabetes among US adults, 2005–2012. Diabetes Care*.* 2017;40(7):887-893. 28373205 10.2337/dc16-1509 PMC5481991

[27] **Liu C, Foti K, Grams ME, Shin J-I, Selvin E.** Trends in self-reported prediabetes and metformin use in the USA: NHANES 2005–2014. J Gen Intern Med*.* 2020;35:95-101. 31637644 10.1007/s11606-019-05398-5 PMC6957593

[28] **Chiou TT, Ge Y, Romley JA.** Trends in metformin use among patients with prediabetes: 2008–2020. Diabetes Care*.* 2023;46(1):e1-e2. 36350077 10.2337/dc22-0985

[29] **Richmond II SP, Grubbs V.** How abolition of race-based medicine is necessary to American health justice. AMA J Ethics*.* 2022;24(3):226-232. 10.1001/amajethics.2022.226 35325524

[30] **O'Brien MJ, Vargas MC, Lopez A, et al.** Development of a novel clinical decision support tool for diabetes prevention and feasibility of its implementation in primary care. Prev Med Rep*.* 2022;29:101979. 36161117 10.1016/j.pmedr.2022.101979 PMC9501986

[31] **Kandula NR, Moran MR, Tang JW, O’Brien MJ.** Preventing diabetes in primary care: providers’ perspectives about diagnosing and treating prediabetes. Clin Diabetes*.* 2018;36(1):59-66. 29382980 10.2337/cd17-0049 PMC5775003

[32] **Tseng E, Greer RC, O’Rourke P, et al.** National survey of primary care physicians’ knowledge, practices, and perceptions of prediabetes. J Gen Intern Med*.* 2019;34:2475-2481. 31502095 10.1007/s11606-019-05245-7 PMC6848700

[33] **O’Brien MJ, Moran MR, Tang JW, et al.** Patient perceptions about prediabetes and preferences for diabetes prevention. Diabetes Educ*.* 2016;42(6):667-677. 27621093 10.1177/0145721716666678 PMC5228412

[34] **McGowan BM, Bruun JM, Capehorn M, et al.** Efficacy and safety of once-weekly semaglutide 2· 4 mg versus placebo in people with obesity and prediabetes (STEP 10): a randomised, double-blind, placebo-controlled, multicentre phase 3 trial. Lancet Diabetes Endocrinol*.* 2024;12(9):631-642. 39089293 10.1016/S2213-8587(24)00182-7

[35] **Jastreboff AM, le Roux CW, Stefanski A, et al.** Tirzepatide for obesity treatment and diabetes prevention. N Engl J Med*.* 2024. 10.1056/NEJMc2504728 40561545

[36] **Tseng E, Durkin N, Clark JM, Maruthur NM, Marsteller JA, Segal JB.** Clinical care among individuals with prediabetes in primary care: a retrospective cohort study. J Gen Intern Med*.* 2022;37(16):4112-4119. 35237886 10.1007/s11606-022-07412-9 PMC8890680

[37] **Mainous III AG, Rooks BJ, Wright RU, Sumfest JM, Carek PJ.** Diabetes Prevention in a US Healthcare System: A portrait of missed opportunities. Am J Prev Med*.* 2022;62(1):50-56. 34736802 10.1016/j.amepre.2021.06.018

